# Validation of reference gene stability for normalization of RT-qPCR in *Phytophthora capsici* Leonian during its interaction with *Piper nigrum* L.

**DOI:** 10.1038/s41598-024-58139-y

**Published:** 2024-03-27

**Authors:** Saranya Vijayakumar, Manjula Sakuntala

**Affiliations:** 1 Plant Disease Biology Lab, Rajiv Gandhi Centre for Biotechnology, Thiruvananthapuram, 695014 India; 2 Research Centre, University of Kerala, Thiruvananthapuram, India

**Keywords:** Molecular biology, Plant sciences

## Abstract

The selection of stable reference genes for the normalization of reverse transcription quantitative real-time PCR (RT-qPCR) is generally overlooked despite being the crucial element in determining the accuracy of the relative expression of genes. In the present study, the stability of seven candidate reference genes: actin (*act*), α-tubulin (*atub*), β-tubulin (*btub*), translation elongation factor 1-α (*ef1*), elongation factor 2 (*ef2*), ubiquitin-conjugating enzyme (*ubc*) and 40S ribosomal protein S3A (*ws21*) in *Phytophthora capsici* has been validated. The validation was performed at six infection time points during its interaction with its susceptible host *Piper nigrum*, two developmental stages, and for the combined dataset. Four algorithms: geNorm, NormFinder, BestKeeper, and the ΔCt method were compared, and a comprehensive ranking order was produced using RefFinder. The overall analysis revealed that *ef1*, *ws21*, and *ubc* were identified as the three most stable genes in the combined dataset, *ef1*, *ws21*, and *act* were the most stable at the infection stages, and, *ef1*, *btub*, and *ubc* were most stable during the developmental stages. These findings were further corroborated by validating the *P. capsici* pathogenesis gene *NPP1* expression. The findings are significant as this is the first study addressing the stability of reference genes for *P. capsici–P. nigrum* interaction studies.

## Introduction

*Phytophthora capsici* Leonian is a plant pathogen belonging to the oomycete class under the kingdom Chromista^1^. This oomycete pathogen poses a threat to various important crops including tomato, bell pepper, eggplant, cucumber, watermelon, melon, squash, broad beans, common beans, runner beans, and, strawberry^2–5^. It has been reported to infect plants from 49 different botanical families^6^. The pathogen has a worldwide occurrence and a unique life history owing to its genetic heterozygosity and causes different symptoms depending on the host, plant part infected, and environmental conditions. *Phytophthora capsici* is a hemibiotroph with an initial biotrophic phase in which the host tissues show no signs of infection and a later necrotrophic phase in which the symptoms become visible as it induces necrosis of host tissues^1^. *P.capsici* being a heterothallic species, in the presence of the two mating types A1 and A2, determined by their ability to produce acyclic diterpene mating hormones α1 and α2, respectively, is capable of producing sexual spores called oospores. These oospores possess the ability to remain dormant in the soil for a long time^7^. This oomycete pathogen can also produce asexual structures called zoosporangia which release swimming zoospores under favourable conditions, aiding in the spread of the pathogen to new environments or host plants^8^. The zoospores germinate into mycelia which represents the vegetative growth stage composed of branching filaments (hyphae)^2^.

Black pepper (*Piper nigrum* L.) is one of the most widely traded spices globally and holds significant economic value. Native to the Western Ghats of India, it is currently grown in several tropical regions worldwide, including India, Vietnam, Indonesia, Brazil, and Malaysia^9^. These countries are major producers and exporters of black pepper. Black pepper has a long history and has been highly valued for its culinary and medicinal properties. *Phytophthora* foot rot has consistently been recognized as the primary obstacle to black pepper production among the seventeen reported diseases^10^. It can infect almost all parts of *P. nigrum*—the roots, stem, leaves, and fruits and the pathogen can cause significant damage and yield losses in black pepper plants^11^. It thrives in warm and humid conditions, making it particularly problematic in tropical and subtropical regions where black pepper is commonly cultivated. The disease can spread rapidly through water, soil, and plant-to-plant contact, making it challenging to control and manage^12^. The quality and yield of black pepper can be significantly compromised due to *P. capsici* infection, leading to economic losses for growers. The disease initiates as dark brown spots appearing on the young leaves located in the lower region of the bush. These spots quickly expand, eventually covering a significant portion of the leaf. The affected leaf spots exhibit distinct fimbriate margins, and as a result of the infection, the leaves drop prematurely. Additionally, this pathogen can infect the green stems and branches, leading to rotting. In the case of root rot, the infection begins in the fibrous root system, progresses towards the main root, eventually reaches the collar or base of the bush^13^. Management of *Phytophthora* blight involves a combination of cultural practices, chemical control, biological control, and the use of resistant plant varieties^14^. However, the effectiveness of these measures may vary, and studies in this regard are still very naïve. Recently, effector biology has been identified as an emerging trend in the field of phytopathology. Effectors are proteins secreted by pathogens that manipulate the host plant’s defenses and promote infection. Understanding the role and function of these effectors is crucial for developing effective strategies to protect crops from *Phytophthora* infections^15^.

Plant-pathogen interaction studies often utilize omics techniques like transcriptomics to unravel the intricate molecular mechanisms involved^16^.However, when it comes to validating the RNA-seq data or examining the expression levels of defense-related genes in hosts and pathogenic determinant genes at different infection time points, Reverse Transcription-quantitative real-time Polymerase Chain Reaction (RT-qPCR) remains the preferred approach^17–19^. While RT-qPCR is commonly employed, obtaining reliable and meaningful results relies on several crucial factors, including sample preparation, nucleic acid extraction techniques, and meticulous assay execution. In the realm of relative RT-qPCR experiments, the choice of reference genes (RGs) for normalization assumes paramount importance. The identification of stable RGs contributes to the robustness and accuracy of RT-qPCR analyses. According to the MIQUE (Minimum Information for Publication of Quantitative Real-Time PCR Experiments) guidelines, normalizing RT-qPCR data against a single RG is no longer acceptable. Moreover, the suitability of RGs may vary depending on the specific host–pathogen system, experimental setup, tissue, or cell types^20^. Therefore, it is crucial to select and validate appropriate RGs tailored to the unique requirements of each study.

Currently, no published studies have investigated the stability of RGs specifically in *P. capsici*. In most gene expression studies related to *P. capsici*, commonly used RGs such as actin, tubulin, or RGs validated in other *Phytophthora* species^21–23^ have been employed^24–26^. Therefore, it is necessary to validate the stability of RGs to be used for the normalization of RT-qPCR in *P. capsici*. Thus, the present study aims to validate the stability of seven RGs that are commonly used in *Phytophthora* species—actin (*act*), α-tubulin (*atub*), β-tubulin (*btub*), translation elongation factor 1α (*ef1*), elongation factor 2 (*ef2*), ubiquitin-conjugating enzyme (*ubc*), and 40S ribosomal protein S3A (*ws21*)—for selecting the most suitable RGs of *P. capsici* for specific experimental purposes. This validation will encompass two developmental stages (mycelia and zoospores), multiple infection time points (1.5hpi, 3hpi, 6hpi, 12hpi, 24hpi, and 48hpi), as well as the combined dataset. The integration of multiple algorithms like geNorm^27^, NormFinder^28^, ΔCt^29^, BestKeeper^30^, and RefFinder^31^ to give a comprehensive ranking, will contribute to a more reliable evaluation of RG stability, enhancing the accuracy of gene expression analysis and interpretation in *P. capsici*.

## Results

### RNA quality assessment

The quality of RNA used in this study was assessed to ensure an accurate estimation of gene expression. The NanoVue spectrophotometer measured the 260/280 absorbance ratio of RNA samples, yielding values ranging from 1.9 to 2.1. This indicated that the RNA samples were of good purity. The integrity of RNA was evaluated through denaturing gel electrophoresis. The bands corresponding to 18S rRNA and 28S rRNA were visually observed. The presence of sharp and distinct bands in the gel indicated intact, undenatured RNA molecules, signifying high RNA integrity (Supplementary Fig. S1).

### Assessment of amplification efficiency and specificity

**Figure 1:**
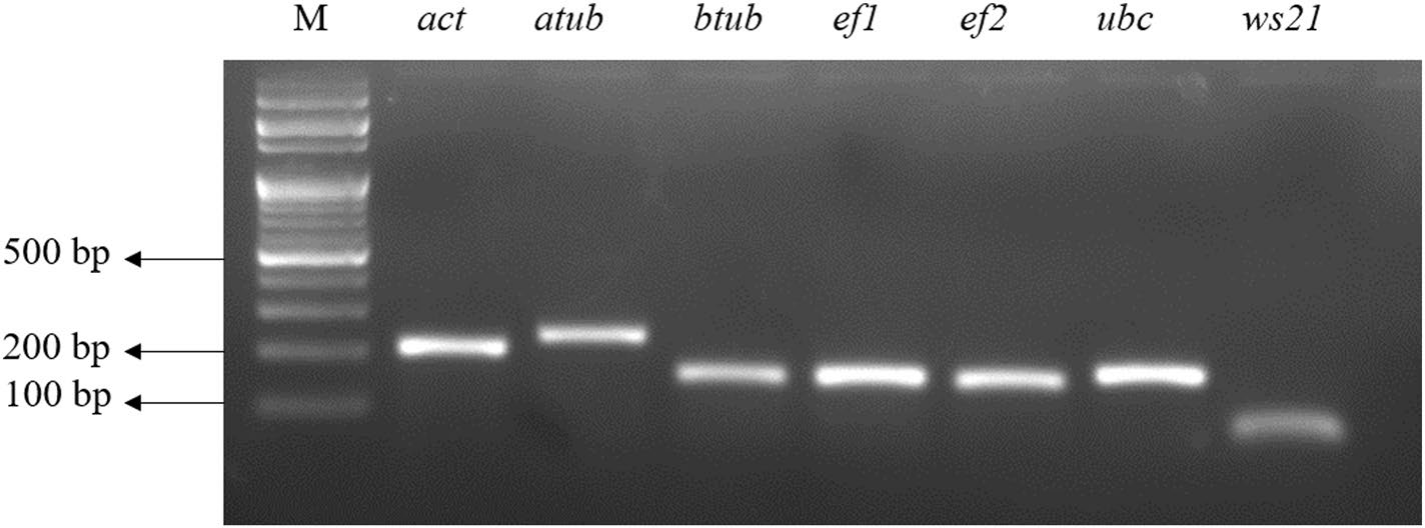
Agarose gel analysis of amplified PCR products from candidate reference genes. This figure displays the 2% agarose gel image depicting the amplified products of the expected size obtained from the primer pairs of candidate reference genes- actin (*act*), α-tubulin (*atub*), β-tubulin (*btub*), translation elongation factor 1α (*ef1*), elongation factor 2 (*ef2*), ubiquitin-conjugating enzyme (*ubc*), and 40S ribosomal protein S3A (*ws21*). The label “M” corresponds to the 100 bp DNA ladder. For uncropped gel image see Supplementary Fig. S4.

The amplified products from each primer pair showed a single band with the expected amplicon length (Fig. 1). Melt curve analysis further indicated a single peak suggesting the absence of primer dimer formation (Supplementary Fig. S2). Additionally, the no template control displayed no detectable signal. Table 1 provides a summary of gene name, protein ID, amplicon length, primer sequences, and melting temperature (Tm). The slope of the calibration curve was used to compute the amplification efficiency (Supplementary Fig. S3). The efficiency percentage ranged from 96.85% (*ef1*) to 109.33% (*act*) (Table 2). The correlation coefficients ranged between 0.991 (*btub*, *ef2*) to 0.999 (*act*) (Table 2).

**Table 1:** List of reference genes used in the study along with the corresponding primer pairs, amplicon length and melting temperature (Tm).

Gene name^a^	Gene symbol	Protein ID^a^	Primer sequence (5′–3′)	Amplicon length (bp)	Tm (^°^C)
Actin	*act*	132086	Forward: ACTGGGACGACATGGAGAAG Reverse: GCCAGAGGCATACAGGGATA	206	89
α-tubulin	*atub*	530856	Forward: TGCGTGAAGTCATCTCCATC Reverse: TGCGTACTTCGTCACACACA	236	90
β-tubulin	*btub*	126822	Forward: CCAGCTTCAGCCTTCACTTC Reverse: CATCCCAATCCTGATCCTGT	158	85
Translation elongation factor 1-α	*ef1*	510859	Forward: AGGTTCACATTTCGCTGGTC Reverse: CAGGCGTACTTGAACGAGGT	160	87
Elongation factor 2	*ef2*	511,907	Forward: CGTAACATGTCCGTGATTGC Reverse: GATGGTAATGCAACGCTCCT	153	89.5
40S Ribosomal protein S3A	*ws21*	503,425	Forward: GAAGCGGATCAACAAAGAGC Reverse: GCCGGGAAGTAGATGTTCAG	165	91.5
Ubiquitin-conjugating enzyme	*ubc*	510705	Forward: AAGGATGAGGACCAGGCTTT Reverse: CGAAGGCCTCAATCAGAGTC	151	87.5

^a^Gene names and protein IDs as given in JGI genome portal.

**Table 2:** Parameters regarding slope, efficiency and correlation coefficient as obtained from RT-qPCR.

Gene symbol	Slope	Efficiency (%)	R^2^
*act*	− 3.117	109.33	0.999
*atub*	− 3.138	108.307	0.994
*btub*	− 3.159	107.265	0.991
*ef1*	− 3.4	96.854	0.998
*ef2*	− 3.363	98.33	0.991
*ws21*	− 3.285	101.573	0.993
*ubc*	− 3.33	99.668	0.993

### Expression stability of candidate reference genes

**Figure 2:**
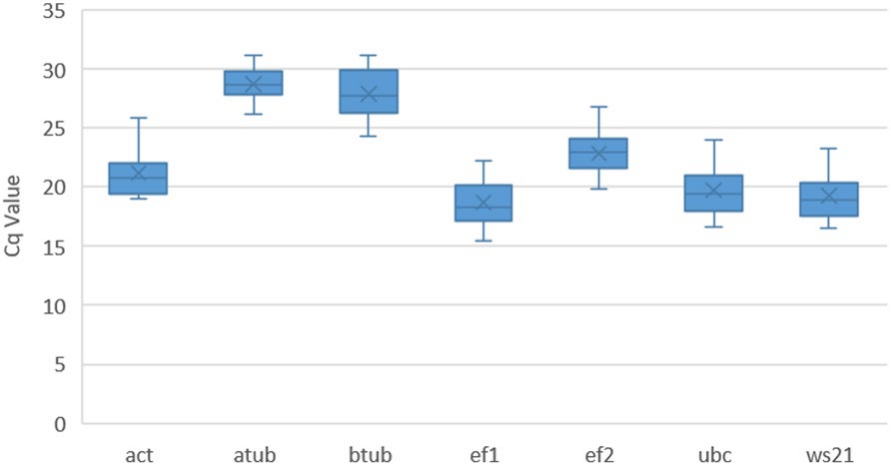
Variation in C_q_ values across all the cDNA samples. This box plot graph illustrates the variation in C_q_ values for seven candidate reference genes (actin (*act*), α-tubulin (*atub*), β-tubulin (*btub*), translation elongation factor 1α (*ef1*), elongation factor 2 (*ef2*), ubiquitin conjugating enzyme (*ubc*) and 40S ribosomal protein S3A (*ws21*) in the combined dataset which includes all the cDNA samples used in the present study. The central line within a box represents the median value, while the lower and upper boxes depict the 25th and 75th percentiles, respectively. The mean value is indicated by the cross inside the box and the whiskers extends to the maximum and minimum values observed.

The mean C_q_ (quantification cycle) values of all seven genes from three biological groups were utilized for the estimation of stability across the three datasets (Supplementary Table 1). The candidate RGs showed a large variation in mean C_q_ ranging from 15.5 (*ef1*) to 31.1 (*atub*) while considering the total dataset (Fig. 2).

### ΔCt method

In the ΔCt method, the average of standard deviation is used as a measure of variation within a set of ΔCt values for a particular RG across different samples or experimental conditions. The RG with the lowest average standard deviation is considered more stable because it exhibits less variation in expression across the samples or conditions. Table 3 provides the average of standard deviations and ranking of RG across various datasets. In all three datasets, *ef1* emerged as the highest-ranked gene. On the other hand, *atub* exhibited the least stability, both in the infection and combined dataset.

**Table 3:** Expression stability rankings of seven candidate reference genes across three experimental conditions as calculated by geNorm, NormFinder, BestKeeper, ΔCt and RefFinder analysis.

Condition	Rank	geNorm		NormFinder		BestKeeper		ΔCt		RefFinder	
		Gene	Stability	Gene	Stability	Gene	Stability	Gene	Stability	Gene	Stability
Infection dataset	1	*ef1,ubc*	0.575	*ef1*	0.296	*ef1*	0.984	*ef1*	0.68	*ef1*	1.5
	2	*ws21*	0.617	*ws21*	0.486	*btub*	0.966	*ws21*	0.76	*ws21*	2.78
	3	*act*	0.684	*ubc*	0.51	*ubc*	0.954	*Ubc*	0.78	*act*	3.13
	4	*ef2*	0.727	*act*	0.58	*ws21*	0.952	*Act*	0.8	*ubc*	3.46
	5	*btub*	0.767	*btub*	0.616	*ef2*	0.949	*Btub*	0.83	*ef2*	3.83
	6	*atub*	0.806	*ef2*	0.735	*act*	0.938	*ef2*	0.89	*btub*	4.79
	7	*–*	–	*atub*	0.745	*atub*	0.885	*Atub*	0.91	*atub*	5.12
Developmental dataset	1	*ef1,btub*	0.196	*btub*	0.27	*ubc*	0.999	*ef1*	0.83	*ef1*	1.68
	2	*ubc*	0.268	*ef1*	0.281	*ef1*	0.999	*Btub*	0.87	*btub*	2.06
	3	*ws21*	0.506	*ubc*	0.534	*btub*	0.994	*Ubc*	0.9	*ubc*	2.71
	4	*act*	0.596	*ws21*	0.68	*ws21*	0.986	*ws21*	1.05	*ef2*	4.3
	5	*atub*	0.908	*act*	0.884	*act*	0.986	*Act*	1.11	*ws21*	4.47
	6	*ef2*	1.127	*atub*	1.223	*atub*	0.978	*Atub*	1.46	*atub*	4.56
	7	*–*	–	*ef2*	1.53	*ef2*	0.916	*ef2*	1.67	*act*	5.14
Combined dataset	1	*ef1,ubc*	0.62	*ef1*	0.236	*ef1*	0.991	*ef1*	0.82	*ef1*	1.5
	2	*ws21*	0.649	*btub*	0.529	*ubc*	0.97	*ws21*	0.91	*ws21*	2.91
	3	*act*	0.683	*ws21*	0.556	*btub*	0.97	*Btub*	0.93	*ubc*	3.13
	4	*btub*	0.751	*ubc*	0.684	*ws21*	0.965	*Ubc*	0.97	*btub*	3.18
	5	*atub*	0.928	*act*	0.77	*act*	0.94	*Act*	1.02	*atub*	4.14
	6	*ef2*	1.023	*atub*	1.061	*ef2*	0.853	*ef2*	1.26	*act*	4.16
	7	*–*	–	*ef2*	1.08	*atub*	0.851	*Atub*	1.26	*ef2*	4.74

### NormFinder

NormFinder uses a model-based approach to estimate the variation of gene expression data. It evaluates the stability of potential RGs by analyzing the variation between sample groups (intergroup variation) and within sample groups (intragroup variation). The algorithm calculates a stability value for each reference gene, with a lower value indicating more stable expression across different conditions or sample groups. Based on the NormFinder analysis results, presented in Table 3, the genes *ef1*, *btub*, and *ws21* demonstrated the highest stability in the combined dataset. However, in terms of the infection dataset, *ef1*, *ws21*, and *ubc* exhibited the greatest stability, while *btub*, *ef1*, and *ubc* ranked as the most stable genes during the developmental dataset. Conversely, *ef2* displayed the least stability in combined as well as developmental datasets.

### BestKeeper

In BestKeeper analysis, several descriptive statistics like arithmetic mean (AM), geometric mean (GM), minimum (Min) and (Max), standard deviation (SD), coefficient of correlation (r), and coefficient of variance (CV) are used to evaluate the stability of RGs. The BestKeeper index is calculated using a geometric mean of the cycle threshold (Ct) values of potential reference genes^30^. We used the r value in BestKeeper to rank the genes. The closer the r value is to one, the greater the stability of the gene^32^. In the infection dataset and combined dataset, BestKeeper identified *ef1* as the most stable gene, followed by *btub*, *ubc*, and, *ws21*. Conversely, *atub* was ranked as the least stable. In the developmental dataset, BestKeeper revealed a high correlation among *ubc*, *ef1*, and *btub* (Table 3).

### geNorm

**Figure 3:**
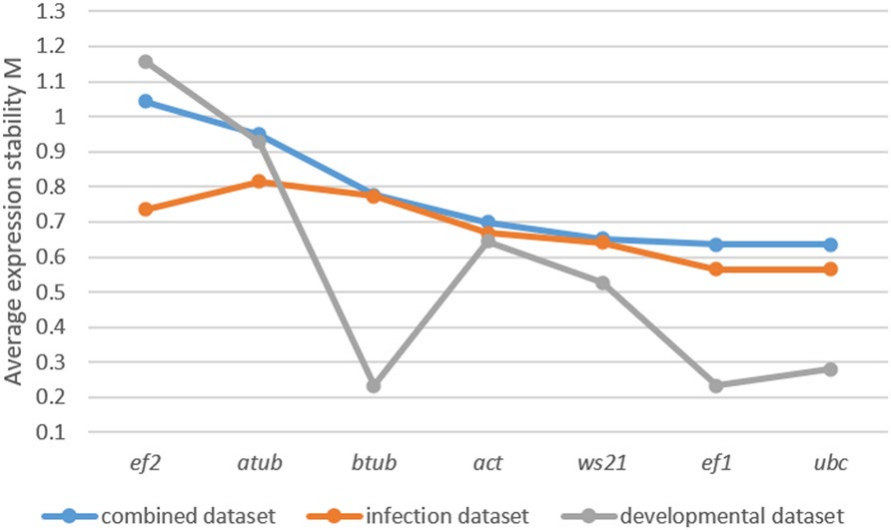
Expression stability ranking by geNorm algorithm. This figure presents the results of expression stability analysis based on average expression stability M value determined by geNorm analysis in the three datasets.

geNorm operates on the principle that the expression ratio of two ideal internal control genes remains consistent across all samples, irrespective of experimental condition or cell type. This software calculates a gene stability measure called the M value for each candidate RG. The M value represents the average pairwise variation between a gene and all other genes in the dataset. Genes with lower M values are considered more stable^27^. In both the combined and infection datasets, geNorm identified *ef1* and *ubc* as equally stable genes followed by *ws21* and *act* in the second and third ranking positions. In the developmental dataset, *ef1* and *btub* were ranked as the top genes, followed by *ubc* and *ws21.* The genes *ef2* and *atub* were the least ranked in the combined and developmental datasets, while *btub* and *atub* were given the least ranking in the infection dataset (Table 3 and Fig. 3).

**Figure 4:**
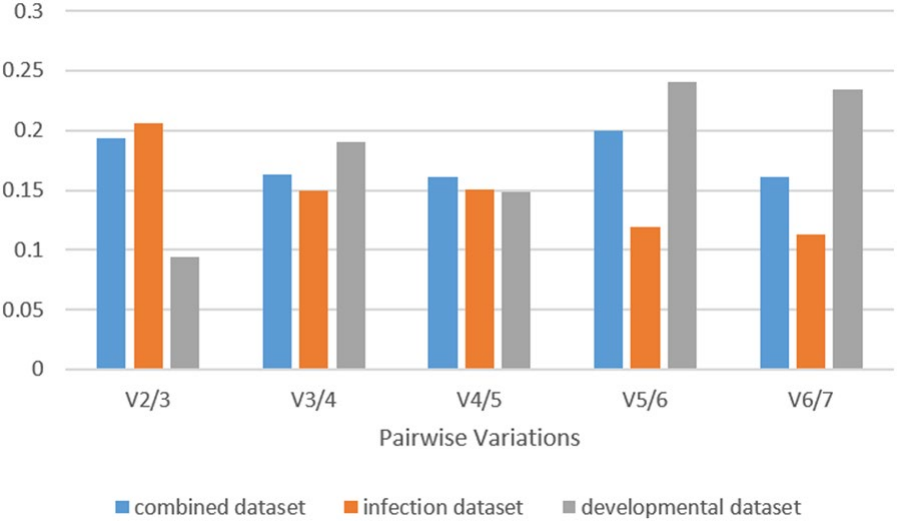
Determination of optimal number of reference genes by geNorm analysis. Pairwise variation (V_n_/V_n+1_) analysis performed by geNorm algorithm to determine the optimal number of reference genes for normalization. This analysis involved calculating the variation between the normalization factors NF_n_ and NF_n+1_.

geNorm also calculates pairwise variation (V_n_/V_n+1_), which measures the effect of adding an additional reference gene on the normalization factor. The default cut-off value for pairwise variation in geNorm is typically set at 0.15. This means, if the pairwise variation value (V) between two sequential combinations of RGs falls below this cut-off, it suggests that adding an additional RG does not significantly improve the stability of the normalization factor. As mentioned in the geNorm manual, the authors of geNorm do provide this default cut-off value as a guideline, but it is not a fixed rule and can be adjusted based on the particular experimental context. In the developmental dataset, the pairwise variation value for V_2/3_ was found to be low at 0.094 (Fig. 4). This indicates that the addition of a third control gene is not necessary for achieving reliable normalization. However, in the infection dataset, V_3/4_ and V_4/5_ were 0.15, and V_5/6_ was 0.119. These values suggested that including five control genes would be beneficial for achieving reliable normalization in this dataset. For the combined dataset, the lowest pairwise variation value was observed for V_4/5_ which was 0.16 (Fig. 4).

### RefFinder

RefFinder is a comprehensive web-based tool that integrates multiple algorithms, including geNorm, NormFinder, ΔCt, and Bestkeeper, to rank the stability of candidate RG for normalization in gene expression studies. It calculates the geometric mean of the rankings generated by the individual algorithms and generates a consensus ranking that helps to mitigate any potential bias or limitations of a single algorithm^31^. RefFinder analysis yielded almost consistent results with ΔCt, NormFinder, BestKeeper, and geNorm rankings, indicating the stability of RGs across different datasets (Table 3). In both the infection dataset and the combined dataset, RefFinder consistently identified *ef1* as the most stable gene, while the rankings of second and third genes varied slightly among the algorithms. In the combined dataset, *ws21* and *ubc* followed *ef1* in terms of stability, whereas in the infection dataset, *ws21* and *act* were ranked second and third, respectively. In the developmental dataset, RefFinder concurred with ΔCt, NormFinder, BestKeeper, and geNorm outcomes by ranking *ef1*, *btub*, and *ubc* as the top three most stable genes.

### Validation of expression stability

**Figure 5:**
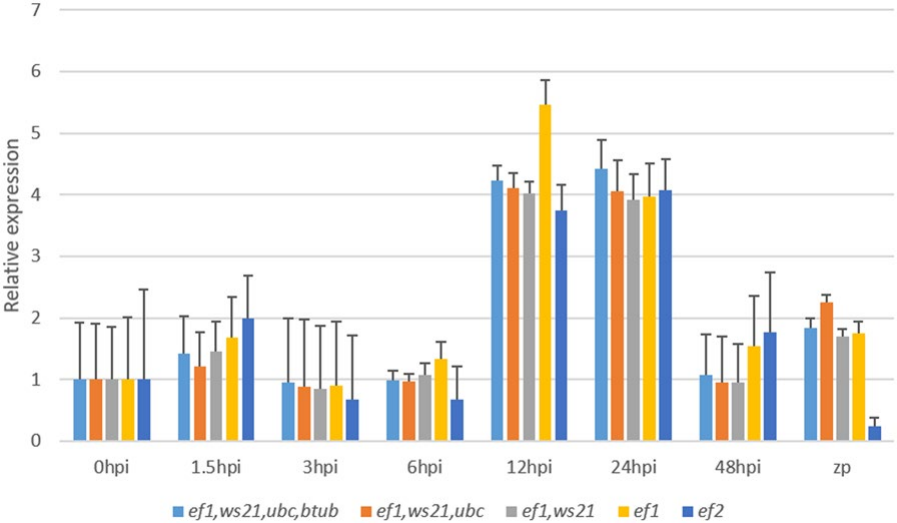
Relative expression levels of *NPP1* gene in the infection time points and in the zoospores sample using 0hpi as control group. Normalization was done against combinations of most stable genes as well as single most stable and least stable gene. The error bars represent standard error.

The *Phytophthora capsici* pathogenicity-related gene *NPP1* was used for the validation purposes. The details regarding the *NPP1* primer are given in supplementary table 2. In the validation process using the target gene *NPP1* in the combined dataset, the comprehensive ranking of candidate RGs suggested by RefFinder was assessed (Fig. 5). Various scenarios were tested, including using a combination of the top four genes (*ef1*, *ws21*, *ubc,* and *btub*), the top three genes (*ef1*, *ws21*, and *ubc*), the top two genes (*ef1* and *ws21*), the highest ranked gene (*ef1*), as well as the least ranked gene (*ef2*) individually. In all the cases where multiple RGs were used, consistent expression patterns were observed, confirming the stability of the selected RGs and emphasizing the importance of employing more than one RG for normalization in RT-qPCR experiments. The results indicated that the expression of *NPP1* peaked from 12 to 24 hpi and decreased by 48hpi. However, when the least stable gene (*ef2*) was used for normalization, particularly in the 48hpi sample and zoospore sample, the relative expression of *NPP1* was misleadingly assessed. Additionally, when a single most stable gene (*ef1*) was used, there was a clear overestimation of gene expression at 12hpi. These findings confirm the stability of selected RGs and underscore the importance of using multiple RGs for normalization in RT-qPCR experiments. By employing a combination of suitable RGs, more reliable and accurate results can be obtained in gene expression analysis.

## Discussion

The RT-qPCR approach is frequently utilized in studies investigating plant-pathogen interactions to gain insights into the underlying molecular mechanisms^33–35^. There is often a concern that despite the widespread use of RT-qPCR, one may not consistently adhere to the recommended guidelines for conducting this experiment, potentially resulting in misleading outcomes^36^. It is widely acknowledged that when determining the relative gene expression of a target gene, it is essential to employ multiple reference genes that exhibit stable expression under the specific biological conditions of interest in order to normalize the data^20^. However, there is a noticeable scarcity of studies focusing on this aspect, particularly in the field of crop plants^37^ and phytopathogens^38^. *Piper nigrum*, commonly known as black pepper is an important non-model spice crop that is susceptible to a highly destructive disease called quick-wilt or foot rot caused by the pathogen, *Phytophthora capsici*^39,40^. The suitability of different RGs in conducting expression studies in the host plant (*P. nigrum*) in response to *P. capsici* infection has been reported earlier^37^. However, to gain a comprehensive understanding of the *P. nigrum*–*P. capsici* phytopathosystem, it is crucial to uncover the molecular undulations of gene expressions associated with the infection steps employed by the pathogen to invade the host plant. Currently, sufficient knowledge in this area is lacking and to address this gap, the present study aims to assess the suitability of seven commonly used RGs in *Phytophthora* species as stable RGs for gene expression studies in *P. capsici*. This study holds the potential to facilitate future research aiming to investigate the expression of pathogenic determinants, such as effectors, in *P. capsici* during its infection of *P. nigrum*. This evaluation was conducted under three specific conditions: (1) six time points during the progression of *P. capsici* infection in *P. nigrum*, (2) at two distinct developmental stages of *P. capsici*, and (3) in the combined dataset. Since gene expression studies heavily rely on the presence of high-quality RNA, thorough care was taken at every step of RNA extraction and handling. Only samples that met the criteria of a 260/280 absorbance ratio above 1.8 and exhibited intact bands of 28S rRNA and 18S rRNA were taken up for subsequent analysis.

The candidate RGs were chosen by reviewing existing literature that discussed the use of RGs in studies related to various *Phytophthora* species. Our extensive search yielded only one study specifically focused on RG selection, and it was conducted on *Phytophthora parasitica*. The study employed the geNorm algorithm to evaluate the stability of selected reference genes across different life stages of *P.parasitica*. The study concluded that *β-actin* and *ef1-α* were not ideal choices, while *β-tubulin*, *ubc*, and *ws21* were more suitable^21^. After this study, several gene expression studies in *P.capsici* have employed the RGs recommended in the aforementioned study in *P.parasitica*, either in combination or individually^26,41–44^. Remarkably, the findings of our study are partially consistent with these results, as *ws21* and *ubc* were identified as stable genes in the combined dataset, while *ubc* and *btub* were also given high rankings in the developmental dataset, according to the geNorm analysis. However, our study also reports *ef1* as the most stable RG across all datasets by geNorm as well as by RefFinder. This contrast in findings emphasizes the need to conduct thorough assessments of RG stability customized to the particular species and experimental conditions under investigation. Notably, *ef1α* was used as an internal control gene in a study on characterization of RxLR effectors in *P. capsici*^45^. Additionally, a study focusing on inhibiting sterol synthesis in *P. infestans* includes the utilization of *ubc*, *ws21*, and *β-tubulin* as selected RGs based on expression stability analysis^46^. In another study investigating *P. infestans* effectors, *ef2α* was utilized as the RG^47^. Furthermore, in a separate study exploring sexual reproduction in *P. infestans*, the authors employed data mining of RNA-seq data to identify PITG_09862, a Kelch domain protein, and PITG_02745, a casein kinase, as top-ranked genes after evaluating their stability with multiple algorithms^23^. In *P.sojae*, eight RGs, namely, *ef1a, ef2a, ws21-1, ws21-2, ubc-1, ubc-2, β-tub,* and *ws41*, were evaluated for their applicability in the study on Argonaute genes. Among these genes, *β-tub* and *ws41* were ultimately utilized in the research^22^. Upon completing the literature survey, we made the decision to assess the stability of the genes actin (*act*), α-tubulin (*atub*), β-tubulin (*btub*), translation elongation factor 1α (*ef1*), elongation factor 2 (*ef2*), ubiquitin-conjugating enzyme (*ubc*), and 40S ribosomal protein S3A (*ws21*) in *P.capsici* as potential RGs for the experimental conditions that were to be studied. After designing the primer pairs, the specificity of the primers were assessed by observing a single band of the expected amplicon size on the agarose gel. Additionally, a melt curve analysis was performed after the PCR, which showed a single peak. To evaluate the PCR amplification efficiency, a calibration curve was constructed, and the efficiency of the PCR was determined by calculating the slope of the curve. Importantly, the calculated efficiency fell within the accepted range of 90–110%, indicating reliable and efficient amplification using all primer pairs.

The stability analysis on RGs was performed using four widely employed methods, namely, geNorm, NormFinder, ΔCt, and BestKeeper. With the exception of BestKeeper in the developmental dataset, all four algorithms consistently ranked *ef1* as the top gene in all conditions. Although there were minor discrepancies in the ranking of the other genes, possibly attributed to variations in the algorithms employed, it was evident from the results that *ws21*, *ubc*, and *btub* consistently held prominent positions. geNorm also calculates the optimal number of RGs to be used for normalizing RT-qPCR data. For our study, geNorm recommends the use of a minimum of two genes in the developmental dataset, and 5 genes in the infection dataset. This is based on a default cut-off value of pairwise variation (V), which is 0.15. However, in the combined dataset, none of the V values were below 0.15, the lowest being 0.16 for V5/6. Taking into account the experimental heterogeneity in the combined dataset, it is important to consider that pairwise variation analysis should be used as a guideline rather than a strict rule^48^. In this case, although using five control genes would be preferable, in general, we recommend using three control genes as a better option than using a single RG for normalization.

RefFinder, a web-based tool that integrates the algorithms of all four methods, and calculates the geometric mean of rankings generated by each algorithm was utilized to get a comprehensive ranking of the RGs. The expression stability ranking according to RefFinder is as follows: in the infection dataset- *ef1* > *ws21* > *act* > *ubc* > *ef2* > *btub* > *atub*, in the developmental dataset- *ef1* > *btub* > *ubc* > *ef2* > *ws21* > *atub* > *act*, in the combined dataset- *ef1* > *ws21* > *ubc* > *btub* > *atub* > *act* > *ef2*. To validate our findings, we selected the necrosis-inducing protein gene *NPP1* (accession number: HM543167) from *P. capsici* which encodes an apoplastic effector protein crucial for the pathogenesis of *P. capsici*. This protein has been observed to induce necrotic responses in the host plant *Nicotiana benthamiana*^25^. Previous research from our lab has shown that *P. capsici* initiates hyphal growth in *P. nigrum* within 6 hpi and proliferates extensively on the leaf, resulting in the formation of progressing necrotic lesions within 24 hpi^40^. Our analysis of *NPP1* gene expression in this study aligns with these findings, revealing a peak expression between 12 and 24 hpi, followed by a decrease by 48 hpi. In summary, our results strongly advocate for the utilization of multiple reference genes tailored to specific experimental conditions and sample types. The selection of these RGs using suitable algorithms is imperative to achieve accurate normalization of RT-qPCR data, thereby ensuring reliable and precise results.

## Methods

### Organisms and inoculation method

Pure culture of *Phytophthora capsici* was originally a gift from Dr. Anith K. Narayanan, Professor and Head of the Department of Agricultural Microbiology at Kerala Agricultural University, Vellayani, Kerala, with due permissions granted for its use in research. The culture was confirmed in our lab by sequencing and maintained at the Plant Disease Biology Lab as *P. capsici* isolate RGCB0451, which was used for the present study. It was routinely cultured in Potato Dextrose Agar (PDA) medium (HiMedia, India) at 28 °C. Pathogen inoculation studies were conducted in the host plant *Piper nigrum* L. variety-Panniyur I, a popular cultivar of Kerala State, India, which was originally procured from authentic stock cuttings maintained at the Agricultural University, Kerala, India. The identity of the plant was confirmed by the Curator of the Herbarium maintained at the Department of Botany, University of Kerala, and subsequently, plants were maintained in the Green house of Rajiv Gandhi Centre for Biotechnology, Trivandrum, Kerala, India. To inoculate *P. capsici*, the second and third leaves of *P. nigrum* L.variety-Panniyur I plants were used. The inoculation followed the method outlined by Ma et al.^24^, with minor modifications. In brief, *P. capsici* was cultured on PDA medium overlaid by a cellophane membrane for easy separation of mycelia^49^. The mycelium, 4 days old, was then placed between two *P. nigrum* leaves and placed on a wet Whatman filter paper in a square petri plate. The plate was sealed with parafilm to maintain humidity. Mycelial samples were collected at 1.5, 3, 6, 12, 24, and 48 h post-inoculation (hpi) for RNA extraction. For the 24 and 48hpi, mycelia were collected along with infected leaf tissues. Additionally, a sample of mycelia was directly collected from a 4-day-old culture medium as a 0hpi sample. The zoospore sample (zp) was harvested following the method described by Granke et al.^50^. Three independent biological replicates were maintained for each sample group.

All plant experiments were performed in adherence with relevant guidelines and regulations.

### RNA isolation

The collected samples were immediately frozen in liquid nitrogen and ground to a fine powder. RNA extraction was carried out using the TRIzol reagent (Invitrogen) according to the manufacturer’s instructions. The concentration and purity of the extracted RNA were determined using NanoVue Spectrophotometer (GE Healthcare, UK). To assess the integrity of the RNA, electrophoresis was performed on a 1.5% agarose-formaldehyde denaturing gel stained with EtBr. Any potential contamination from genomic DNA was eliminated by treating it with DNase (DNA-free, Ambion).

### Selection of candidate genes and primer designing

Through an extensive review of the literature, commonly utilized reference genes in *Phytophthora* species were identified for further investigation in *P. capsici* gene expression studies. These selected genes include actin (*act*), α-tubulin (*atub*), β-tubulin (*btub*), translation elongation factor 1α (*ef1*), elongation factor 2 (*ef2*), ubiquitin-conjugating enzyme (*ubc*), and 40S ribosomal subunit S3A (*ws21*). To design the primers, the Primer 3 Plus (www.bioinformatics.nl/cgi-bin/primer3plus/primer3plus.cgi) bioinformatics tool was employed, utilizing the available *P.capsici* genome sequences from the JGI genome portal (mycocosm.jgi.doe.gov/Phyca11/Phyca11.home.html). The primer specificity was initially validated by conducting PCR amplification and visualizing the products on a 2% agarose gel after electrophoresis. Furthermore, melt curve analysis was performed to confirm the specificity of the primers.

### cDNA synthesis and RT-qPCR analysis

cDNA synthesis was carried out in a 20 μl reaction volume with 1 μg RNA using PrimeScript™ RT reagent kit (TaKaRa, Japan) at 37 °C for 15 min followed by 85 °C for 5 s. Diluted aliquots were used as templates in RT-qPCR assays. RT-qPCR was performed on QuantStudio™ 5 Real-Time PCR system (Applied Biosystems, USA) using TB Green® Premix Ex Taq™ II (Tli RNaseH Plus) kit (TaKaRa, Japan) in a volume of 20 μl consisting of 10 μl TB Green Premix Ex Taq™ II (2×), 0.4 μl ROX Reference Dye II, 2 μl cDNA template, 0.4 μM each of forward and reverse primers and 6 μl nuclease-free water. The RT-qPCR cycling conditions consisted of an initial denaturation step at 95 °C for 10 min, followed by 40 cycles of denaturation at 95 °C for 15 s and annealing at 60 °C for 30 s. To evaluate primer specificity and confirm the absence of primer-dimer formation, a melt curve phase was added after the PCR phase. A technical triplicate for each biological replicate, and no template controls were included in the assay. A standard curve was generated for each primer set using a five-fold dilution series of cDNA.The correlation coefficient (R^2^) and the amplification efficiency (E = 10^(−1/slope)^−1) were calculated from the slope of the regression line in the standard curve by Quantstudio™ Design and Analysis Desktop Software (Applied Biosystems). Primer pairs with slopes ranging from − 3.6 to − 3.1 and efficiency percentage between 90 and 110% with an R^2^ value above 0.99 was chosen.

### Analysis of stability of reference genes

The stability of candidate reference genes was assessed using four commonly used algorithms: geNorm^27^, NormFinder^28^, ΔCt^29^, and BestKeeper^30^. To obtain a comprehensive evaluation of reference gene stability, a web-based tool called RefFinder was utilized, which combines the results from these four algorithms^31^. Raw C_q_ values were directly used as input for ΔCt, BestKeeper, and RefFinder. GeNorm and NormFinder required the conversion of C_q_ values into relative quantity (RQ). Reaction efficiency corrections were included for input values. The stability of all seven genes was assessed in three distinct datasets: (1) the developmental dataset which includes mycelia and zoospores, (2) the infection dataset which includes samples collected at different infection time points (1.5 hpi, 3 hpi, 6 hpi, 12 hpi, 24 hpi, and 48 hpi) and (3) the combined dataset, which included all samples from both developmental and infection conditions.

### Validation of expression analysis using selected reference genes

Various combinations of stable genes were utilized to evaluate the reliability of the chosen reference genes, including the top four, top three, and top two ranked genes, as well as the single most stable gene and the least stable gene. These combinations were employed for normalizing the expression of the target gene *NPP1* (necrosis-inducing protein) in *P. capsici*. The RT-qPCR procedure followed the methods described in the preceding section. The Pfaffl method^51^ was employed to analyze the relative fold change in the *P. capsici* gene *NPP1* after infecting *P. nigrum* leaves and in the zoospore stage with 0hpi sample as the control group.

### Supplementary Information

Supplementary Information.

## Data Availability

All relevant data analyzed during this study are included in this article and in the Supplementary files.
