# Supplementary material for: Validation of reference gene stability for normalization of RT-qPCR in Phytophthora capsici Leonian during its interaction with Piper nigrum L
Source: Sci Rep. 2024 Mar 27;14:7331. doi: 10.1038/s41598-024-58139-y (PMC10973370; doi:10.1038/s41598-024-58139-y)
Supplement: Supplementary file 1 — Supplementary Information. [file 41598_2024_58139_MOESM1_ESM.pdf]

Supplementary information

Validation of reference gene stability for normalization of RT-qPCR in *Phytophthora capsici* Leonian during its interaction with *Piper nigrum* L.

Saranya Vijayakumar[1,2], and Manjula Sakuntala[1,*]

[1] Rajiv Gandhi Centre for Biotechnology, Plant Disease Biology Lab, Thiruvananthapuram, 695014, India

[2]Research Centre, University of Kerala, Thiruvananthapuram, India

[*]smanjula@rgcb.res.in

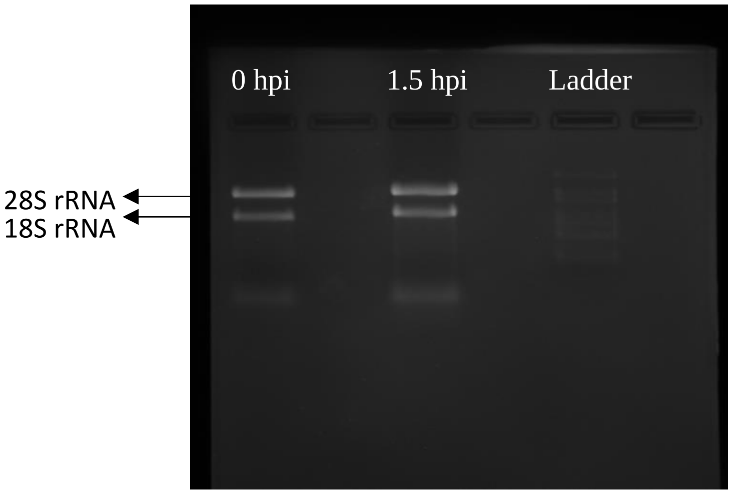

Supplementary Figure S1. RNA gel electrophoresis image showing intact RNA. This figure presents a representative image of 1.5% agarose-formaldehyde RNA gel electrophoresis, showcasing intact RNA. The gel image includes samples from 0hpi and 1.5hpi.

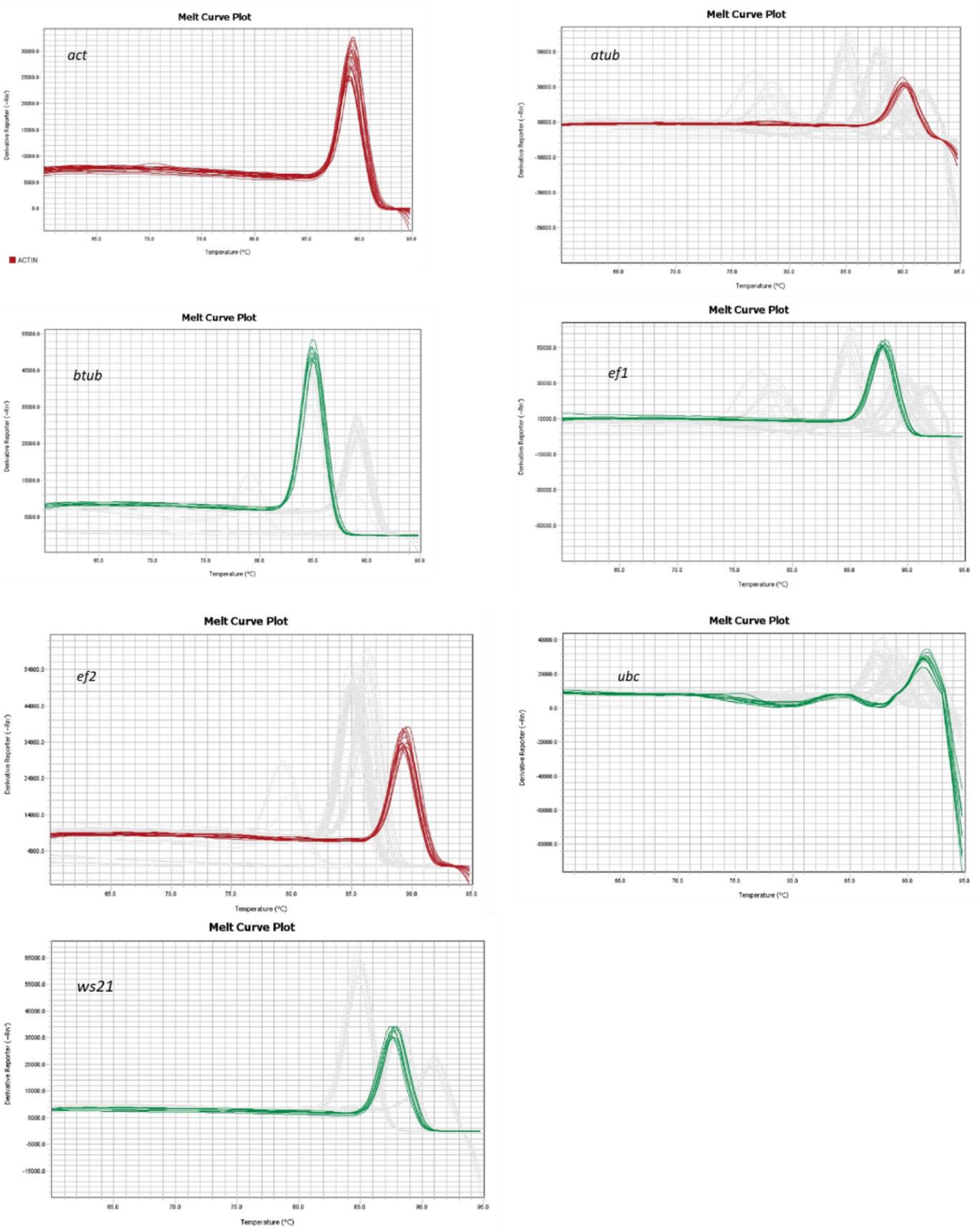

Supplementary Figure S2. Melt curve analysis for candidate reference genes. This figure presents the melt curve analysis results for the tested candidate reference genes (actin (*act*), α-tubulin (*atub*), β-tubulin (*btub*), translation elongation factor 1α (*ef1*), elongation factor 2 (*ef2*), ubiquitin-conjugating enzyme (*ubc*), and 40S ribosomal protein S3A (*ws21*)). The results demonstrate the specificity of primer pairs used.

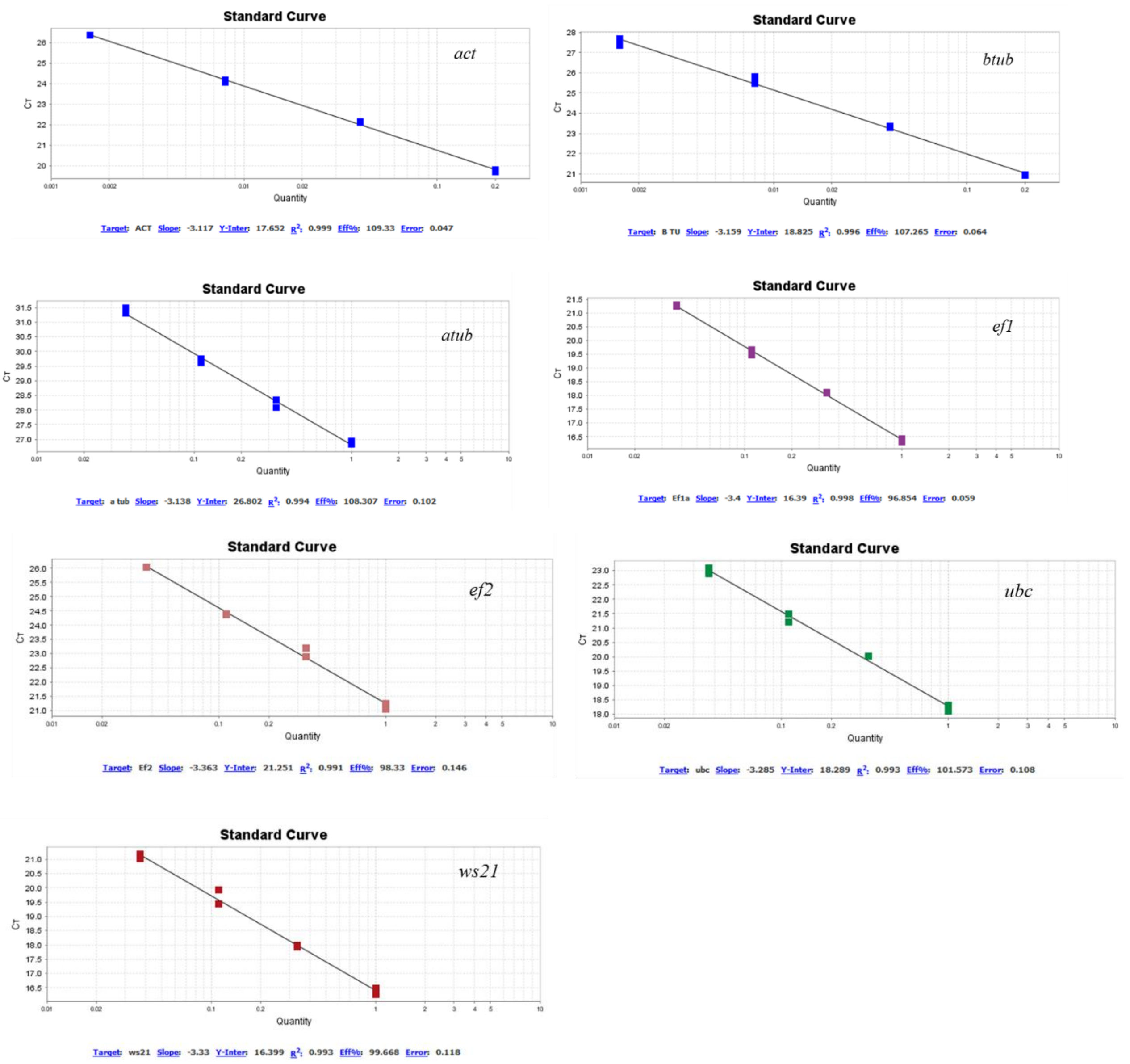

Supplementary Figure S3. Standard curves for candidate reference genes. This figure illustrates the standard curves generated for the candidate reference genes (actin (*act*), α-tubulin (*atub*), β-tubulin (*btub*), translation elongation factor 1α (*ef1*), elongation factor 2 (*ef2*), ubiquitin-conjugating enzyme (*ubc*), and 40s ribosomal protein S3A (*ws21*)). The slope of each standard curve was calculated to determine the amplification efficiency of the reaction for each primer pairs.

sample	*act*	*atub*	*btub*	*ef1*	*ef2*	*ubc*	*ws21*	*NPP1*
0hpi	19.116	26.946	25.303	16.042	19.858	17.677	16.478	27.570
0hpi	19.083	26.587	26.355	17.227	22.823	18.699	17.503	25.823
0hpi	19.293	26.124	24.318	15.511	20.272	17.041	17.754	27.215
1.5hpi	19.480	27.654	26.325	16.708	21.552	17.111	17.399	26.791
1.5hpi	20.173	28.190	27.171	17.580	22.669	18.141	17.528	26.201
1.5hpi	19.014	26.130	25.085	16.003	20.976	16.643	17.096	26.872
3hpi	21.828	30.198	29.751	19.360	23.710	20.109	20.062	31.829
3hpi	22.752	31.173	30.034	20.165	24.506	21.490	20.725	29.086
3hpi	20.768	28.417	26.258	17.356	21.675	18.945	18.711	28.089
6hpi	20.198	28.325	25.154	17.030	19.956	17.507	17.367	27.260
6hpi	19.435	28.088	25.670	16.394	20.477	17.923	17.051	27.018
6hpi	20.999	30.055	26.966	18.260	22.479	18.411	18.364	27.936
12hpi	19.459	27.687	26.803	17.677	21.848	18.925	18.753	26.628
12hpi	19.982	28.600	27.480	18.828	23.043	20.012	18.478	26.452
12hpi	20.734	29.325	28.729	19.648	23.872	19.961	19.452	27.533
24hpi	20.507	28.535	28.331	18.025	22.700	18.599	19.104	26.838
24hpi	21.324	29.243	28.678	19.316	24.068	20.983	19.837	26.906
24hpi	21.356	28.756	27.927	18.335	23.268	20.099	19.790	27.753
48hpi	21.886	29.260	30.405	20.169	25.002	20.075	20.175	31.005
48hpi	22.436	30.549	30.118	21.484	26.786	22.023	20.439	29.783
48hpi	22.075	29.530	29.336	20.385	25.195	20.977	20.646	31.022
zp	24.825	30.293	30.550	21.914	23.937	23.931	23.250	31.967
zp	25.871	29.855	31.147	22.262	24.110	24.019	23.178	31.924
zp	25.871	29.855	31.147	22.262	24.110	24.019	23.178	31.675

Supplementary Table 1. Cq values of candidate reference genes and pathogenicity-related gene. This table presents the mean Cq values of technical triplicates for seven candidate reference genes and a pathogenicity related gene. The values are reported for three biological replicate groups for the specific experimental conditions used in the study.

Gene name	Gene symbol	Accession number	Primer sequence (5'-3')	Amplicon length (bp)	Efficiency (%)	Tm(°C)
Necrosis inducing protein	*NPP1*	HM543167	Forward: GGGTGTCTACGCCTTCATGT<br>Reverse: GGGATTTTCCAGTGAGTCCA	118	102	88

Supplementary Table 2. Details of *NPP1* primer. This table provides details of primer sequence, amplicon length, amplification efficiency (%) and melting temperature(Tm) for the target gene *NPP1*.

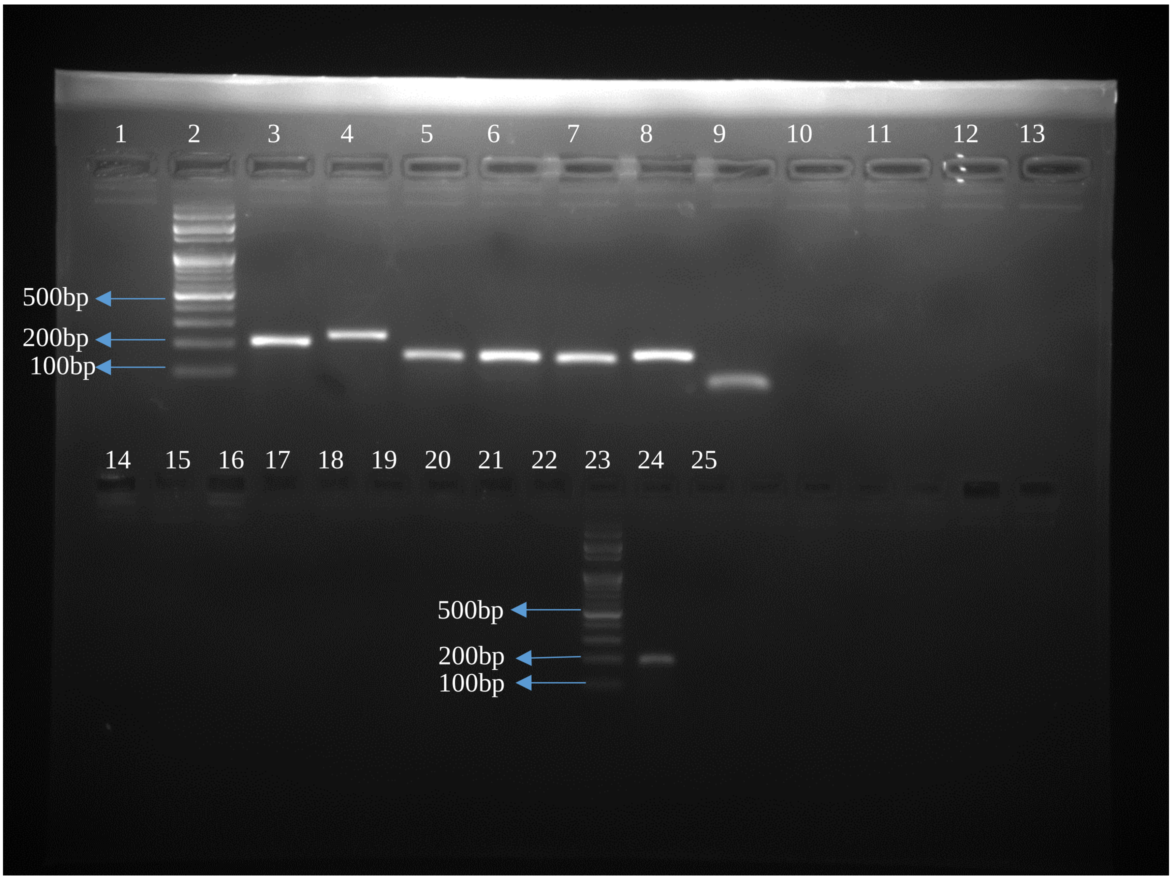

Supplementary figure 4. Agarose gel analysis of amplified PCR products from candidate reference genes. This figure displays the 2% agarose gel image depicting the amplified products of the expected size obtained from the primer pairs of candidate reference genes. The gel lane 3 to lane 9 correspond to the following genes in the order: actin (*act*), α-tubulin (*atub*), β-tubulin (*btub*), translation elongation factor 1α (*ef1*), elongation factor 2 (*ef2*), ubiquitin-conjugating enzyme (*ubc*), and 40s ribosomal protein S3A (*ws21*). The target gene *NPP1* is shown in the lane 24. Lanes 14 to 20 represents the no template controls (NTC) for the seven reference genes and, lane 25 represents the NTC for the *NPP1* gene. Lanes 2 and 23 correspond to 100bp DNA ladder.
